# In Memoriam Frans H. J. Claas

**DOI:** 10.3389/ti.2025.14462

**Published:** 2025-02-27

**Authors:** Dave L. Roelen, Sebastiaan Heidt

**Affiliations:** ^1^ Department of Immunology, HLA Diagnostics Laboratory, Leiden University Medical Center, Leiden, Netherlands; ^2^ Department of Internal Medicine, Erasmus Medical Center Transplantation Institute, University Medical Center Rotterdam, Rotterdam, Netherlands

**Keywords:** histocompatibility and immunogenetics, transplantation, highly sensitized patients, HLA, transplant immunology

Frans Claas, one of the most influential transplant immunologists and histocompatibility experts of his time, unexpectedly passed away on Sunday the 2nd of February 2025, aged 73. He died while on a vacation trip in South Africa with his wife Ilse and dear friends Ronald and Dienne Bontrop.

Frans Claas was born on 6 October 1951 in Eindhoven, a city in the province of North Brabant in the south of the Netherlands. He was born into a very Catholic family, resulting in his first potential career choice of becoming a pope. Alternative career choices were professional football (Frans was goalkeeper at RKVV Tongelre, and almost made it to professional club MVV Maastricht), or biology. After successfully finishing his Gymnasium education in 1970, Frans eventually decided to study biology at Leiden University, for which he took his final exam in 1976. During the last 2 years of his education he was already a student assistant in the laboratory of Jon J. van Rood at the Academic Medical Center in Leiden. After obtaining his biology degree he continued working in this lab and started his PhD studies. He successfully defended his PhD thesis entitled “The Interaction of Drugs and γ-Type Endorphins with Polymorphic Cell Membrane Antigens” on 29 May 1985. Following, he took over the end responsibility of the HLA laboratory, achieving the status of National Reference Center for Histocompatibility Testing. He also became Director of the Eurotransplant Reference Laboratory. On 13 December 1996 Frans became Professor on the Immunogenetics of Transplantation at Leiden University.

Frans was an exceptional scientist, and the true embodiment of the collaborative spirit that has characterized the histocompatibility and immunogenetics field throughout the years. For him, the advancement of science and the wellbeing of patients was always more important than personal benefit or recognition. His pioneering spirit is exemplified by the publication from 1988 where Frans introduced a totally new concept to increase the chance of transplantation for highly sensitized patients [1]. By extensive antibody screening (at that time solely by complement-dependent cytotoxicity (CDC) assays), he showed that it was possible to define “acceptable mismatches” to which a negative crossmatch could be predicted. This work culminated into the still highly successful Eurotransplant Acceptable Mismatch Program [2], in which more than 2000 highly sensitized patients have been transplanted to date.

In his efforts to extend the possibilities for highly sensitized patients Frans became one of the founding fathers of the field of what is now often called “molecular mismatch” analysis. In the early 2000s, Frans teamed up with Rene Duquesnoy, who had just introduced his HLAMatchmaker concept [3]. They showed that additional acceptable antigens for highly sensitized patients could be defined by extrapolating negative CDC antigen reactivity to untested HLA class I antigens by triplet (predecessor of eplet) sharing [4]. Following, his group was the first to show that an increased level of HLA triplet mismatches was associated with an increased chance of *de novo* donor-specific antibody (dnDSA) formation, and that antigen mismatched, but triplet matched transplants did not result in dnDSA formation [5], a finding that is still replicated in studies today. With the transition of triplets to eplets and the start of the HLA Epitope Registry [6], his team made significant contributions to the antibody verification of eplets by developing human HLA-specific monoclonal antibodies [7–9]. His work on differential immunogenicity of HLA mismatches was not limited to solid organ transplantation. His team also explored the role of molecular mismatch in the setting of hematopoietic stem cell transplantation. They showed that HLAMatchmaker analysis was not informative for the cytotoxic T cell precursor (CTLp) frequency [10]. Paradoxically, more amino acid mismatches at the alpha-helices and beta-sheet resulted in less formation of donor reactive CD8^+^ T cells, a finding explained by the necessity of some level of resemblance between mismatched HLA and self-HLA for direct allo-recognition [11]. Linked to these observations were the seminal studies on heterologous immunity, in which cross-reactivity of virus-specific T cells with allogeneic HLA could explain the relatively high frequency of T cells with direct alloreactivity [12, 13].

Frans (Figure 1) was one of the few scientists in histocompatibility that explored the setting of pregnancy for understanding naturally occurring immunological tolerance to a haploidentical situation. Through the years his group explored the unique T cell signature in the human placenta, related to either good or complicated pregnancy outcomes [14–16]. More recent work using mass cytometry highlighted the potential role of myeloid cells in the human placenta [17, 18]. In his research, Frans did not evade controversial subjects, as evidenced by a paper in which a correlation between oral sex and the low incidence of the pregnancy complication preeclampsia was shown, with the hypothesis that soluble HLA could induce immunological tolerance [19].

**FIGURE 1:**
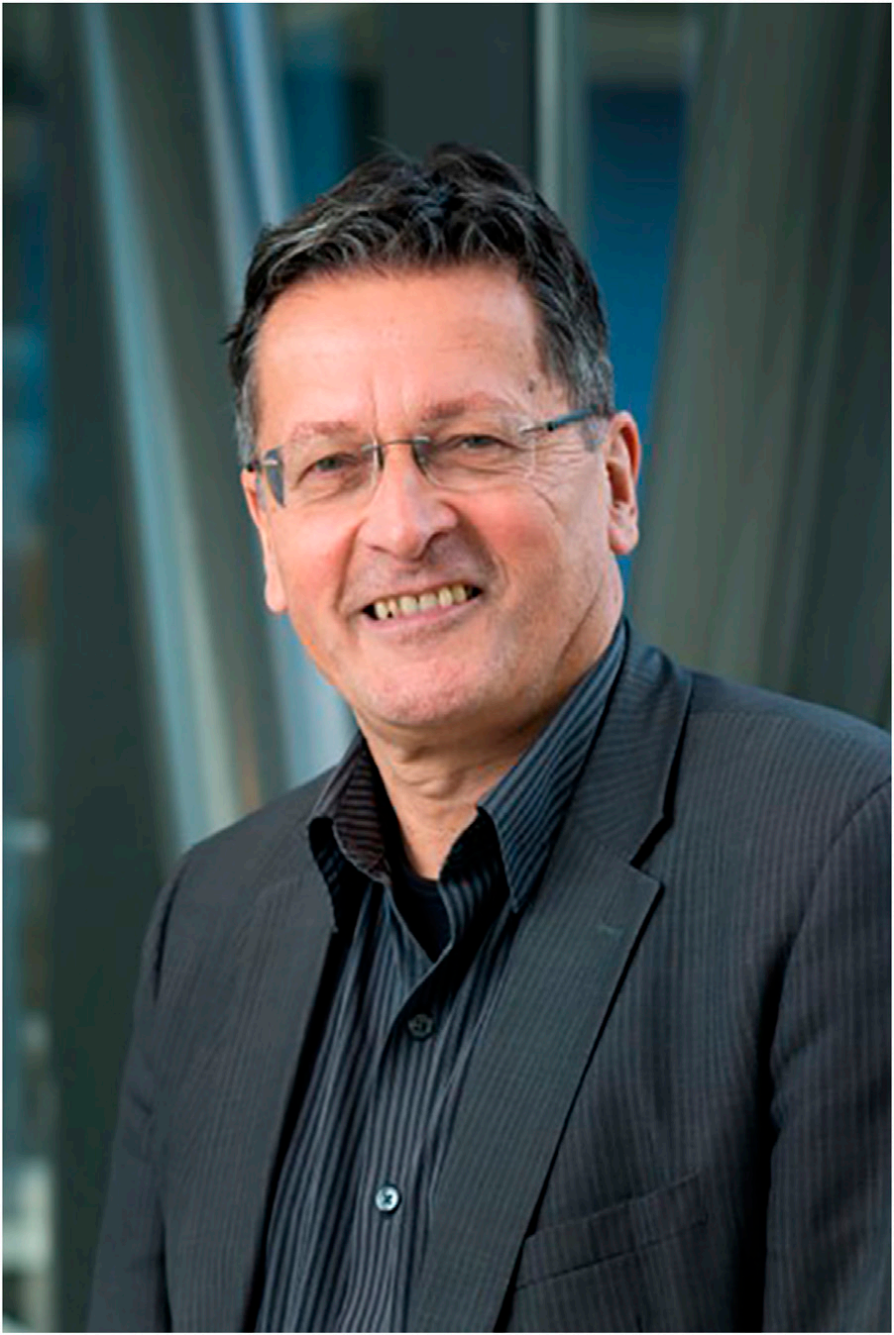
Frans Claas at Leiden University Medical Center.

Frans’ legacy is enormous, with over 600 peer-reviewed papers published. He was member of several advisory committees and consensus meetings [20, 21]. His scientific merits have been recognized by receiving several prestigious awards, including the ASHI distinguished scientist award in 2006, the EFI Ceppellini Award in 2015, and the ASHI Rose Payne Distinguished Scientist Award in 2015. Upon his retirement in 2017 Frans was knighted as a Knight of the Order of the Netherlands Lion by the King of the Netherlands for the impact of his work on society.

Besides his scientific achievements, what his colleagues remember most about Frans is that he was a wonderful human being. He showed interest in everybody, regardless of their knowledge, skillset, or origin. He felt a great deal of responsibility to help scientists from all over the world to improve their knowledge and skills. The lab in Leiden continuously hosted colleagues from all over the world, such as India, Australia, Israel and China, just to name a few. His collaborative spirit was tangible in the lab in Leiden, and beyond. His social skills were second to none, as he took interest in everyone and was always willing to give advice. Moreover, he surely knew how to have a good time. Wherever there was a dance floor, Frans was there to be found. He loved to have a drink with his many friends and talk about science, but also about life outside of science. Frans was an avid runner and completed numerous marathons, with the most notable being the Bordeaux Médoc Marathon, which combined two major passions of Frans.

We hope that his memory will inspire others to selflessly advance science for patient benefit. Finally, we would like to remember Frans by one of his life mottos, “Carpe Diem,” which rings true even more since Frans is no longer with us.

## Data Availability

The original contributions presented in the study are included in the article/supplementary material, further inquiries can be directed to the corresponding author.
